# Risk Assessment for Musculoskeletal Disorders in Forestry: A Comparison between RULA and REBA in the Manual Feeding of a Wood-Chipper

**DOI:** 10.3390/ijerph16050793

**Published:** 2019-03-05

**Authors:** Margherita Micheletti Cremasco, Ambra Giustetto, Federica Caffaro, Andrea Colantoni, Eugenio Cavallo, Stefano Grigolato

**Affiliations:** 1 Department of Life Sciences and Systems Biology, University of Torino, Via Accademia Albertina 13, 10123 Torino, Italy; margherita.micheletti@unito.it (M.M.C.); ambra.giustetto@unito.it (A.G.); 2 Institute for Agricultural and Earthmoving Machines (IMAMOTER), National Research Council of Italy (CNR), Strada delle Cacce, 73, 10135 Torino, Italy; f.caffaro@ima.to.cnr.it; 3 Department of Agriculture and Forest Sciences (DAFNE), Tuscia University, Via San Camillo de Lellis, 01100 Viterbo, Italy; colantoni@unitus.it; 4 Department of Land, Environment, Agriculture and Forestry, Università degli Studi di Padova, Viale dell’Università 16, 35040 Padova, Italy; stefano.grigolato@unipd.it

**Keywords:** ergonomics, forestry, postural risk assessment, RULA, REBA, wood-chipper

## Abstract

The analysis of the postural attitude of workers during the interaction with workstation’s elements and working environment is essential in the evaluation and prevention of biomechanical overload risk in workplaces. RULA (Rapid Upper Limb Assessment) and REBA (Rapid Entire Body Assessment) are the two easiest methods for postural risk assessment in the workplace. Few studies investigated postural risk in forestry sector with regard to human–machine interaction, in particular manually fed wood-chippers. The aim of this study was to evaluate the postures assumed by an operator during the manual feeding of a wood-chipper, and to compare RULA and REBA, in order to identify the more effective and appropriate method for the assessment of the risk of biomechanical postural overload. The results pointed out several postural issues of the upper limbs, and showed that RULA is a more precautionary method to protect operator’s health during the targeted tasks. Implications to improve the human–wood-chipper interaction are discussed.

## 1. Introduction

The analysis of the postural attitude of the worker during the interaction with workstation’s elements and working environment is essential in the evaluation and prevention of biomechanical overload risks in workplaces [1]. Awkward working postures may decrease the workers’ concentration and increase accidents frequency and biomechanical overload [2,3,4,5,6], giving rise to musculoskeletal disorders in the different body regions involved, as at the main limb joints level and the vertebral column [7].

Some standards [8,9,10] dealing with the biomechanical overload caused by incongruous static and dynamic postures have been developed to define the risk assessment methods to evaluate postural load referable to activity and workstation characteristics and to human–machine interaction. They are referenced in international [11] and national [12] legislations aimed at protecting workers’ health and safety.

RULA (Rapid Upper Limb Assessment) [13] and REBA (Rapid Entire Body Assessment) [14] are two easy methods for occupational postural risk assessment. Indeed, previous studies [15,16] showed that observational methods are considered effective in the assessment of biomechanical work-related overload, having the advantage of being more versatile and less expensive in terms of time and resources required compared to objective laboratory measures. Both RULA and REBA allow to obtain a numerical index that represents the quantitative value of the risk at which the worker is exposed during the targeted work activity and to derive the priority level of intervention and the actions needed. The RULA method is suggested for the identification of postural disorders of the upper limbs, of the neck and of the back in relation to the muscular action and external loads applied to the body. The REBA method is applied to identify postural disorders of the whole body, in relation to the muscular action, to the external loads applied to the body and to the type of grip. They are referenced in the international standard for occupational risk assessment [9] and cited among the selected tools for Work-related Musculoskeletal Disorders (WMSDs) prevention according to International Ergonomics Association (IEA) and World Health Organization (WHO) [17]. These methods are also widely applied in several working contexts, mainly industrial work activities (secondary sector) and those producing services and goods (tertiary sector), characterized by a precise standardization of tasks, geometries, gestures and relative execution frequencies that allow a systematic and controlled forecasting and quantification of the biomechanical overload risk. Furthermore, the two methods have been combined and compared to assess postural risk in industry [18,19,20], construction [21], supermarkets [22], hospital and dental sector [23], work with video terminals [24], waste collection activity [25], for firefighters and emergency medical technicians [26], and artisans [27,28,29] and sawmill activities [30].

RULA and REBA have been adopted a few times in the agricultural [31,32] and forestry sectors [33,34] because the evaluation of the biomechanical overload risk in the primary sector activities is more difficult; due to the large variability of the tasks the operators have to perform, depending on crops, operations (seeding, weeding, pruning, harvesting, etc.), the machinery and tools adopted, the, sometimes extreme, weather conditions they have to be carried out with, and the daily and seasonal exposure, as well as the lack of a strict standardization of the work in general [35,36]. Interaction with machines, tools and environments in agriculture, and particularly in forestry, requires therefore a particular attention in the application of risk assessment methods conceived for other contexts, or the formulation of specific methods that consider the distinctive characteristics of these activities [37].

Kundu and Gaur [38] compared RULA and REBA in a study addressed at investigating how much these techniques were appropriate for evaluating the postures assumed by agricultural workers. They highlighted some shortfalls in using these techniques to study risk factors associated with agricultural field operations and suggested to add some factors as posture duration, field condition, environmental factors and nutritional status to better assess the occupational risks and possible remedies, especially for agricultural work in the fields.

Besides RULA and REBA, other methods have been used to assess the postural risk in manual agricultural activities. For example, Kong et al. [39] compared RULA, REBA, OWAS (Ovako Working Posture Analyzing System [40]) and ALLA (Agricultural Lower Limb Assessment [41]) for various agricultural tasks. They found that ALLA better estimated the risk because it identifies critical issues that the other methods do not analyse and, therefore, it always returns a higher level of risk, compared to the other methods. Ojha and Kwatra [42] combined REBA and VAS (Visual Analog Scale) methods in rice cultivation manual operations. REBA indicated postural load and suggested interesting corrective measures. However, in both studies [39,42], the object of the analysis was a manual activity, while, in developed countries, most of the farming operations require the use of machinery and workers spend many hours in interacting with them, making the investigation of the human–machine interaction more relevant [43].

Among the studies specifically dealing with the human–machine interaction in agriculture, RULA is the most frequently adopted method. Vyavahare and Kallurkar [44] used RULA to assess postures assumed during the interaction with agricultural machines such as maize dehusker-sheller. The study analysed and evaluated the risk of various key postures, such as squat, forward/lateral bending, hands flexion/extension, wrist/spine twisting. Putri et al. [45] used RULA to study the use of thresher machines for threshing rice plants. In the first study, RULA helped to optimize a digital human manikin posture, resulting in precise risk assessment and better designed and widely accepted products and workplaces, while, in the second study, RULA provided indications to redesign the machine to reduce injuries and musculoskeletal overload caused by the mismatch between the dimensions of the engine and farmers’ postures and dimensions.

In the forestry sector, the studies report postural risk evaluation mainly referred to manual tools. Gallo and Mazzetto [34] applied different methods including REBA and OWAS to evaluate the postural risks from the cutting operations with chainsaw, showing their good applicability for the assessment of WMSD in forestry. The comparison between OWAS and REBA showed that REBA has a higher level of detail of assessment because it considers the angles between body segments and extremities as the wrist, the neck, the elbow and the shoulders (parameters that are not considered by OWAS method) for both sides of the body and, furthermore, it assesses the type of handle coupling and the characteristics of the performed activities. REBA results to be suitable for suggesting interventions to be performed to decrease musculoskeletal overload, even though it has not been developed specifically for forestry operations and it presents some weaknesses during the assessment, as the lack of coded postures as the kneeling posture.

Forestry is recognized as a highly hazardous industry [46] and working postures are one of the most investigated risk factors for workers’ safety, even though the postural aspects in the human–machine interaction have been under investigated in this sector [47,48]. With regard to machinery, manually fed wood-chippers are one of the most widely used machines in forestry, agricultural, landscaping and urban tree maintenance to reduce the volume of woods for following disposal or re-use in bio-energy production [47]. The attention to this machine is rising because of the significant increasing interest in biomass production as biofuel [49] and because they are often involved in fatal and non-fatal injuries [50]. Data on the occurrence of accidents provide an objective index of the danger of machinery as well as a valid reason for identifying the most critical features of these machines.

In the United States, between 1992 and 2002, Struttman [50] reported 31 fatal and 2042 non-fatal accidents involving wood-chippers. The estimated social cost of these fatal accidents were estimated in 2003 to US$28.5 million; furthermore, an in-depth analysis disclosed that 58% of these fatal accidents involved groundskeepers and machinery operators in the forestry and agricultural sectors [50]. Analyses of the non-fatal accidents showed that 60% of the accidents caused immediate injury or amputation of parts of the upper-body limbs. For 25% of these injuries, the victims were unable to return to work for periods of up to 30 work days [51]. Further studies indicated that more than one-third of these accident victims had less than 11 months of experience in that particular job [50].

In the further period between 2008 and 2018, statistics from the United States Occupational Safety and Health Administration of Department of Labor [52] reported 56 incidents during chipping operations: 64% of the accidents dealt with feeding operations and 36% of them were fatal for the operators. From the same statistics, the accidents not related with feeding operations of wood-chippers had a lower rate, 16%, of fatal accidents.

Accurate statistics about accidents with wood-chippers are not available from most of the European Union countries [53]. Only the French Ministry of Agriculture, Agrifood, and Forestry reported that at least one severe accident related to the use of wood-chippers occurs every year in France and that, in most of the cases, the operators involved were young apprentices [54], one of the category of users for which warnings to eliminate hazardous behaviours when intrinsically safe machine are used, are less effective [55,56].

Based on the literature review, the aim of this study was to identify the more effective and appropriate method between RULA and REBA for the assessment of the risk of biomechanical postural overload, evaluating the postures assumed by an operator during the manual load of a wood-chipper, in a controlled experimental setting. The study further discusses which of the two methods is more precautionary in evaluating postural risk and reports higher risk indices, for the specific activity considered.

For the present study, a small manually fed wood-chipper has been selected since: (1) small-size wood-chippers are widespread among forestry, agricultural and urban green maintenance operators; (2) similar types of wood-chippers are used for occasional and accessory operations to the main activity [47]; (3) often experienced, but not specialized, operators are involved in the wood-chipping operations and this category of operators are known to be the most exposed to safety risks [57]. As a consequence, the manual feeding seems to be critical both in terms of safety and health risks for the operators [58,59].

## 2. Materials and Methods

### 2.1. The Wood-Chipper

The manually fed wood-chippers generally consist of a feeding system, a chipping units based on rotating knives (drum or disc mounted) and on a discharge system. Alternative solutions for the comminution devices are available for production of quality wood chips for fuel [60]. In the case of manually-fed wood-chippers, the operators feed small trees, part of trees and/or branches into the infeed chute by hand with a following risk of biomechanical postural overload, which is typical of manually-loaded wood machines [61].

**Figure 1:**
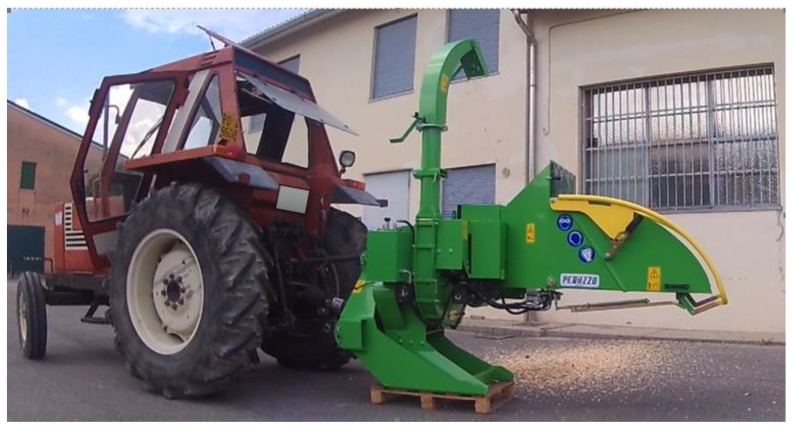
The manually fed wood-chipper used during the test.

The study was carried out with a manually fed wood-chipper model Tirex, made available by the manufacturer Peruzzo (Peruzzo Srl, Curtarolo, Italy). The machine was connected to the rear three-point hitch of a 55 kW tractor and was powered by the rear Power Take-Off (PTO) (Figure 1). The feeding system consists of a feeding hopper with an infeed chute above 600 mm from the ground level and with a width of 1200 mm at the external edge. The feeding system consists of two horizontal rollers electro-hydraulically controlled by a load-limiting mechanism and located at the end of the infeed chute at a distance of 1200 mm from the external infeed chute limit, in accordance with EN 13,525:2005 + A2:2009 [62]. The machine is equipped with an electro-hydraulic feed control bar fixed on the bottom and along the two sides of the infeed chute. The chipping unit consists of a flywheel (diameter 620 mm, mm thickness 30 mm) rotating at 1500 rpm and with a torque of about 33 Nm. The flywheel is equipped with four knives (thickness 25 mm and length 200 mm) while an anvil is fixed to the frame of the machine. The manufacturer claims the machine is able to chip wood stems with a diameter up to 180–200 mm.

### 2.2. Research Design

**Figure 2:**
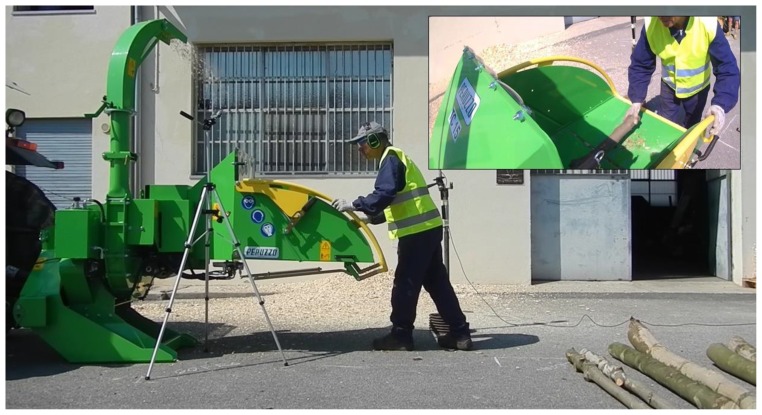
Example of tasks to be performed during the tests.

The study was conducted at the Azienda agraria sperimentale “L. Toniolo” of the Università degli Studi di Padova (Padova, Italy). Since we were interested in controlling as many variables as possible to better compare RULA and REBA, the evaluation of the differences in risk levels between the two methods was carried out by keeping the machine and participant characteristics constant, while changing the log size. To reduce the inter-individual variability in the adoption of the targeted postures, one single participant was involved in the study. He was a not-specialized operator, with no previous history of either occupational accidents or musculoskeletal disorders. Specific measures were defined for the logs to be used in the tests, with regard to log length and diameter, and an acceptable mass, ranging between 2 and 10 kg, to be manually handled by the operator (Figure 2).

Plane-tree wood was used for the tests, preparing both logs of different length and diameters, and branches of more variable dimensions, in order to observe if and how much the size of the wood induced different postures and gestures, which may be associated with different levels of postural risk. The logs were prepared in three different lengths (1 m, 1.5 m, 2 m) and two diameters (65 mm and 135 mm). For each length/diameter combination and for the branches, three elements were prepared to perform three repeated tests. Therefore, six tasks involving logs plus one task regarding branches were performed. The operator performed each task three times to evaluate any intra-individual difference in postures and gestures with respect to the manipulated element. Thus, overall seven tasks were performed for a total of 21 loadings (six tasks for the logs, each repeated three times, and one task for the branch repeated three times). The 21 loadings were randomly performed.

The research protocol was approved by the Research Advisory Group of the Institute for Agricultural and Earthmoving Machines (IMAMOTER) of the National Research Council of Italy (CNR) on 28 February 2018.

### 2.3. Video Recording

The operator was video recorded and photographed while feeding the machine. One video camera was placed in front of the infeed chute while a second one recorded images from the side of the infeed chute. During the analysis of the recorded videos, every time a log or a branch was loaded, the frame-by-frame vision was carried out until the most critical position was detected. The video analysis was performed with Kinovea software, an open-access video analysis software available online reproducing the video in slow-motion. Kinovea is a valid and reliable method to perform motion and postural analysis [63]. It allows for detecting body and its districts’ angles during the single posture observations: angles were measured according to the reference axes reported in RULA and REBA methods to obtain the corresponding score.

### 2.4. Assessment Methods of Applied Biomechanical Risk

The study investigated the human–chipper interaction comparing two observational methods of biomechanical overload postural risk: RULA method [13] and REBA method [14].

In RULA method [13], the body is divided into different parts gathered into two groups, A and B: group A includes arm, forearm and wrist of the right and left limb; group B includes the neck, the trunk and the feet. The method consists in assigning a score to each segment depending on the posture taken and it allows to obtain two distinct scores (Scores A and B) through the use of numerical tables or spreadsheets; these scores represent the level of postural load of the musculoskeletal system, determined by the combination of the postures of the whole body. Muscle use and force scores are then added to Scores A and B to obtain two new scores (Scores C and D) that, through a third table or a spreadsheet, allow for obtaining the final score, or Grand Score.

Based on the appropriate combination of scores, the final score can range between 1 and 7. The final score is related to four levels of action and four levels of risk. Scores, actions and risk levels for RULA are summarized in Table 1:

- Action level 1: Low risk level. A score of 1 or 2 indicates that posture is acceptable if it is not maintained or repeated for long periods.
- Action level 2: Medium risk level. A score of 3 or 4 indicates that further investigation is needed and changes may be required.
- Action level 3: High risk level. A score of 5 or 6 indicates that investigation and changes are required soon.
- Action level 4: Very high risk level. A score of 7 indicates that investigation and changes are required immediately.

In REBA method [14], the body is divided into different segments divided into two groups: the first one includes neck, torso and legs; the second group is composed by arm, forearm and wrist without distinction from the right or the left one. The method consists in assigning a score to each segment of the body according to the posture taken and, using numerical tables or spreadsheet, it allows for obtaining two different scores that represent the level of postural load of the musculoskeletal system determined from the combination of the whole body postures. The two scores should be respectively added to the grip score and to the load and strength score to get two new scores (Scores A and B). From the use of a third table or spreadsheet, it is possible to obtain Score C, which, added to the activity score, allows for obtaining the final score, or Grand Score. The final score can range between 1 and 15 and it is related to five levels of action and five levels of risk. Table 1 reports scores, actions and risk levels for REBA:

- Action level 0: The risk is negligible so no action is required.
- Action level 1: Low risk level. The final score between 2 and 3 indicates that changes may be required.
- Action level 2: Medium risk level. The final score from 4 to 7 indicates the need for measures and further analysis.
- Action level 3: High risk level. The final score between 8 and 10 indicates the need for an intervention and a change in a short time.
- Action level 4: Very high risk level. The final score from 11 to 15 indicates that an action is immediately required.

### 2.5. Data Analysis

For each task, three risk indices were obtained for each of the two methods, RULA and REBA.

The goal was to analyze the highest level of risk calculated, considering the variability of the execution of the gesture, evaluated three times separately. Among the results obtained, the highest value, among the three for each task, and then the highest between the right and left side was selected.

The results of the two scores calculated for each task with RULA and REBA were compared to highlight any tendency of any of the two methods to over or underestimate the risk. This was done both on the basis of risks category, considering the action level—color code (Table 1), and the values of normalized indices.

In order to compare the differences between REBA and RULA scores, it was necessary to normalize the absolute values as the two methods are based on different scales: RULA has four risk levels that categorize scores from 1 to 7, while REBA is based on five levels of risk that categorize scores from 1 to 15. The use of standardized scores to compare different methods for postural risk assessment has been adopted previously in similar analyses [64,65], thus we normalized the score values ranging from 0 to 1, applying the following formula to each worst value obtained for each task by the two methods:

normalized value = [score obs − score value min]/[score value max − score value min].

## 3. Results

The risk assessment carried out by applying RULA and REBA (Table 2) showed a medium-high level of risk for all the tasks. None of the tasks reported a score referable to a negligible or low risk as well as a very high risk level that would require an immediate intervention.

Regarding the differences between the risk indices of each task, it can be seen that, based on the dimensional differences of the handled logs, the risk level is limited in the case of logs with longer length and smaller diameter. This could be due to the posture induced by small length logs which could cause a higher postural overload because of the greater proximity to the machine that could determine worse joint angles. The highest indices were observed in the manipulation of short and larger diameter logs (Task 2), while the lower levels of risk indices occurred in the manipulation of the branches (Task 7). The comparison between the results obtained with RULA and REBA, based on the resulting action level, highlighted a good overlapping between the two methods, with the exception of Task 3, related to wooden elements of an intermediate size (1.5 m) and small diameter (65 mm). In this case, the RULA carried out a more precautionary assessment as it gave an index that fell into a higher action level compared to the corresponding level calculated with REBA. In the comparison of the normalized numerical indices, all of the RULA scores were higher than the corresponding REBA scores for all of the tasks (Table 3). The application of RULA showed to be more precautionary than REBA for all the tasks, as it returned a higher score value in each loading.

## 4. Discussion

The present study showed that RULA and REBA are two effective methods for assessing the postural overload determined by incongruous postures adopted in the act of manually loading the wood-chipper with logs of various diameters and lengths. The two methods appeared to be both effective and suitable for the identification and quantification of the level of postural risk using a manually fed wood-chipper, as they highlighted similar levels of urgency of intervention, analysis and modification actions. The high congruence between RULA and REBA evaluations confirmed similar results from other investigations in the industrial context [66]. However, the present study showed that RULA tended to be more precautionary, giving a higher risk index, for all the tasks, consistent with previous studies comparing the two methods in the industrial sector [18]. This underestimation by REBA was also found in KOSHA’s research [67] in different sectors like ship building, automotive, electronics, general manufacturing, and service industries.

Comparing the action level in which the calculated indices fall, and, therefore, the corresponding severity and urgency of intervention, RULA and REBA suggested an almost identical level and, in only one case (Task 3, intermediate size and small diameter wooden elements), RULA returned a higher action level than REBA (Table 2). Task 3 is also the one which reported the highest difference between normalized RULA and REBA scores. Comparing normalized scores, RULA always resulted in being more precautionary as it returned higher values for all the tasks. The evaluation of the branches’ manipulation task presented the most contained difference between the two normalized values, but also in this case the risk estimated by RULA, even if slightly, prevailed. RULA appeared to be constantly more precautionary in the risk assessment for all of the tasks considered, both in medium or high risk conditions. RULA also seemed to be more precise as it better highlighted the differences in the level of risk exposure between the manipulation of logs and branches. This could be due to the presence of different postural issues of the upper limbs, such as radial or ulnar deviation of the wrist, rotation of the wrist and movements performed across the body or out to the side, which play an important role in the postural risk assessment of these tasks and are not considered by REBA. About the branches loading, RULA normalized score returned a value of 0.33, which was the lowest of all tasks. REBA did not seem to be influenced by these differences among logs and branches manipulation. The minimum normalized REBA score was 0.29, and it was the same value for both the manipulation of the branches (Task 7) and for the manipulation of logs of Tasks 3 and 5.

RULA highlighted how biomechanical workload in the interaction with the wood-chipper is limited to the upper part of the body and it determines an important involvement especially of the wrist and forearm, and on the rachis due to flexion of the chest and postural asymmetries. REBA scores, obtained evaluating the lower limb biomechanical risk in greater detail, had no higher values than RULA in all the tasks, confirming the absence of serious criticalities for the lower limbs in wood-chipper manual feeding.

According to the results of the present study—even though they were obtained by observing one single operator and should not therefore be considered as conclusive—RULA is more precautionary, probably because of the more precise workload evaluation of the upper limbs and trunk. RULA considers in a much more limited way the postural and workload conditions of the lower limbs compared to REBA, but, nevertheless, in the present investigation, REBA did not give higher risk estimation values, which means that, during the loading of the wood-chipper, there was not any evidence of risk to the lower limbs (otherwise, REBA would have highlighted it with a higher risk index). Therefore, RULA may be a more adequate method for the assessment of postural risk of interaction with the machinery when exposure to risk of the lower limbs is less relevant. This consideration confirms results from previous studies which reported the effectiveness of RULA in evaluating the interaction with agricultural machines [44].

The analysis pointed out different RULA scores due to the different lengths and diameters of logs, with shorter ones determining incongruous postures. This consideration opens new perspectives for the analysis of human–wood-chipper interaction stressing the importance of avoiding short logs during the cutting operations, when they are intended to be chipped. In this way, a postural disadvantage occurs even if the mass is reduced. Future investigation should be addressed at investigating a larger number of operators regarding how much log mass, besides the length, determines incongruous postures (high RULA score) and physical effort (applied force and perceived fatigue). The results of such studies could provide suggestions about an “optimal” cutting length for logs, determining a better postural conditions for operators of manually fed wood-chippers. Indeed, in ergonomics, it is not enough to concentrate on the design of the machine, but it is important to intervene in the different components of human–machine interaction through a holistic approach, as widely documented in the literature and recommended by international standards [68,69,70,71].

Besides the attention paid to safety and technical characteristics of the forestry machine, this study highlighted the importance to inform and train the operators to perform gestures, postures and activities in accordance with the ergonomic principles. In this way, in addition to optimize the human–machine interaction, it can be possible to intervene on the organization and management of the whole activity. This integrated approach may contribute to risk reduction and enhance the system productivity and operator’s wellbeing.

## 5. Conclusions

Agricultural and forestry operators interact with a variety of tasks and machinery, which can require ad hoc methods for the assessment of postural risk. The present investigation showed that the RULA method is suitable for the evaluation of postural overload in the human–machine interaction related to a manually fed wood-chipper, more than the REBA method, because it showed indices that corresponded to a higher level of risk for all the tasks observed, independently of the shape, size, mass of the wooden material and, therefore, it would be a more precautionary method to protect operator’s health. Further considerations about the postural risk of manually-loaded wood-chippers should focus not only on the posture of the operators, but also on the safety standards imposed by the safety regulations (for instance, the former EN 13,525:2005 + A2:2009 [62]). The extended application of RULA in real and differentiated agro-forestry conditions will allow for assessing postural risks and physical effort to improve human–machine interaction and operators’ wellbeing in the wood-chipping activity.

## Figures and Tables

**Table 1:** RULA and REBA scores with the respective action level.

RULA Score	Action Level	REBA Score	Action Level	Risk Level
		1	0	Negligible
1–2	1	2–3	1	Low
3–4	2	4–7	2	Medium
5–6	3	8–10	3	High
7	4	11–15	4	Very High

RULA: Rapid Upper Limb Assessment; REBA: Rapid Entire Body Assessment.

**Table 2:** Comparison between RULA and REBA risk evaluation (worst case for each task calculated with each method) for the seven tasks considered with details of the dimensional characteristics of the manipulated wooden elements.

Task ^1^	Object Length (m)	Object Diameter (mm)	Object Mass (kg)	RULA Worst Case	REBA Worst Case	Action Level
1	1	65	2–10	5	8	both 3
2	1	135	2–10	7	11	both 4
3	1.5	65	2–10	5	5	3 with RULA 2 with REBA
4	1.5	135	2–10	6	10	both 3
5	2	65	2–10	4	5	both 2
6	2	135	>10	5	8	both 3
7	Branches			3	5	both 2

^1^ Note. Tasks 1–6 represent the loading of logs with three different lengths (1 m, 1.5 m, 2 m) and 2 diameters (65 mm and 135 mm); Task 7 represents the loading of branches.

**Table 3:** Comparison between the normalized scores of RULA and REBA indices for the seven tasks.

Task ^1^	RULA	REBA
1	0.67	0.50
2	1.00	0.71
3	0.67	0.29
4	0.83	0.64
5	0.50	0.29
6	0.67	0.50
7	0.33	0.29

^1^ Note. Tasks 1–6 represent the loading of logs with different lengths and diameters; Task 7 represents the loading of branches.
